# Genetic Background of Hypertension in Connective Tissue Diseases

**DOI:** 10.1155/2020/7509608

**Published:** 2020-02-03

**Authors:** Bogna Grygiel-Górniak, Iwona Ziółkowska-Suchanek, Elżbieta Kaczmarek, Mariusz Puszczewicz, Natalia Rozwadowska

**Affiliations:** ^1^Department of Rheumatology and Internal Diseases, Poznan University of Medical Sciences, Poznan 61-545, Poland; ^2^Institute of Human Genetics, Polish Academy of Sciences, Poznan 60-479, Poland; ^3^Department of Bioinformatics and Computational Biology, Poznan University of Medical Sciences, Poznan 60-806, Poland

## Abstract

Peroxisome proliferator-activated receptors (*PPAR gamma-2*) and beta-3-adrenergic receptors (*ADRB3*) are involved in the risk of hypertension. But their exact role in blood pressure modulation in patients with connective tissue diseases (CTD) is still not well defined. In this study, 104 patients with CTD and 103 gender- and age-matched controls were genotyped for Pro12Ala and C1431T polymorphisms of the *PPAR gamma-2* gene and Trp64Arg polymorphism of the *ADRB* gene. Anthropometric and biochemical measurements were evaluated, followed by genotyping using TaqMan® SNP genotyping assays and polymerase chain reaction-restriction fragment length polymorphism. The prevalence of analyzed genotypes and alleles was comparable between patients with CTD and the control group, as well as hypertensive and normotensive subjects. Patients with CTD have lower body fat and higher body water amount, serum glucose, and triglyceride (TG) levels. Hypertensive subjects are older and have higher body mass, BMI, waist circumference (WC), body water content, glucose, and TG concentration. The multivariate analysis revealed that hypertensive subjects with Ala12/X or Trp64Trp have higher body mass and WC when compared to normotensive subjects. Trp64Trp polymorphism was also characterized by a higher TG level, while T1431/X subjects had higher WC. The presence of CTD, visceral fat distribution, and increased age are the predictors of hypertension development. Hypertensive patients with CTD and Trp64Trp polymorphism have an increased risk of visceral obesity development and metabolic complications, which in turn affects the value of blood pressure. In addition, either Ala12/X or T1431/X predicts the visceral body fat distribution in hypertensive subjects.

## 1. Introduction

Two genes, peroxisome proliferator-activated receptor gamma (*PPAR gamma-2*) and beta-3-adrenergic receptor (*ADRB3*), are involved in metabolic and inflammatory processes. *PPAR* belong to the nuclear hormone receptor superfamily, from which the PPAR gamma-2 isoform plays a key role in regulating adipogenic differentiation and glucose homeostasis [1, 2]. Two genetic variants of *PPAR gamma-2*—Pro12Ala (rs1801282) and C1431T (SNP rs3856806)—are related to metabolic disorders [3]. Pro12Ala is associated not only with an increased risk of obesity, insulin resistance, and diabetes mellitus [1, 4–8] but also with an increased risk of cardiovascular diseases including hypertension [9–11]. The second polymorphism of *PPAR gamma-2* (C1431T) is also related to metabolic and cardiovascular risk; however, the data are inconsistent [6, 7, 12–14]. Some studies show that the T1431 allele is significantly associated with a reduced risk of coronary artery disease [15], while others indicate an increased risk of myocardial infarction in diabetic patients [6]. The role of C1431T polymorphism is also underlined in hypertension and obesity [13, 14, 16, 17]. Similar to *PPAR gamma-2*, the Trp64Arg polymorphism of the *ADRB3* gene (e.g., Trp64Arg) influences metabolic parameters and cardiovascular risk [12].

Since the ratio between polymorphisms of *PPAR gamma-2* and *ADRB3* genes is not well established in hypertensive patients with CTD, we tested whether analyzed genetic factors are associated with blood pressure values and metabolic parameters in this group. Therefore, we determined the frequency of the analyzed variants and polymorphisms of the *PPAR gamma-2* gene in CTD patients, and we investigated their association with hypertension in the context of anthropometric and biochemical parameters.

## 2. Materials and Methods

### 2.1. Study Group

In this study, 111 patients were selected from patients in the Department of Rheumatology and Internal Diseases. The participants were also selected from our previous study [18]. Those patients with severe kidney and liver diseases, with infections, with untreated thyroid disorders, that are nonsmokers, with skin ulcerations during CTD, and without supplementation of minerals and vitamins were selected to the study. Thus, 104 subjects meeting the above criteria were included for further analysis. Average body mass-matched healthy controls were enrolled onto the study. Of the 104 patients, nearly 70% with CTD required treatment orally with glucocorticosteroid. All hypertensive patients with CTD used blood pressure-lowering medications. Informed consent was signed by each patient. Research was conducted according to the principles expressed in the Declaration of Helsinki, and signed consent was obtained from each patient. The study was approved by the local research ethics committee (Bioethics Committee of Poznan University of Medical Sciences, no. 791/15).

### 2.2. Anthropometric Measurements

Basic anthropometric parameters included body mass (measured in underwear) and height measurements. The waist was measured on the midline between the lowest part of the 12th rib and the suprailiac crest by the WHO method, and hip circumferences were measured at the widest point over the buttocks [19]. BMI was calculated as weight divided by height squared (kg/m^2^), and the waist hip ratio (WHR) was estimated as waist circumference to hip circumference. A bioimpedance analyzer (Bodystat 1500, Bodystat Ltd., UK) was used to assess fat content as a proportion of total body mass. The bioimpedance analysis was performed with a single frequency (50 kHz) device.

Each subject was examined at 8:00 AM in a controlled environment at room temperature (RT). After 20 min of rest in a supine position, brachial SBP and DBP were determined as the average of three measures obtained by an experienced medical staff member on the patient's nondominant arm and following a 10 min rest using a standard mercury sphygmomanometer (the mean of three measurements of SBP and DBP was calculated). Blood pressure was measured according to the guidelines of the European Society of Hypertension Working Group on BP Monitoring [20]. The diagnosis of HT was given if systolic blood pressure exceeded 140 mmHg and/or diastolic blood pressure was higher than 90 mmHg. The specific characteristics of pulsatile arterial hemodynamics included the analysis of two components of blood pressure: mean arterial pressure (MAP) and pulse pressure (PP) [21]. PP was determined by subtracting the diastolic from the systolic blood pressure, and MAP was calculated by using the formula: MAP = [(SBP + 2 × DBP)]/3 [21, 22].

### 2.3. Blood Parameter Measurements

Blood samples were drawn from the antecubital vein after an overnight fast and were collected in tubes containing EDTA. Serum samples were separated from clotted blood (15 min, RT) and centrifuged (15 min, 3000 × *g*). Enzymatic colorimetric assays (Pentra 400, Horiba ABX) were used to measure glucose and lipid profiles (total cholesterol (TC), high-density lipoproteins (HDL) and low-density lipoproteins (LDL), and triglycerides (TAG)). Samples were immediately centrifuged, and serum was separated and directly used for assays. The serum level of LDL was determined using the Friedewald equation [LDL‐C TC {HDL‐C (TG/5)}] [23].

### 2.4. Genetic Evaluation

A detailed description of the methodology was included in our previous studies [2, 18, 24]. DNA samples from patients and controls were isolated from peripheral blood lymphocytes with a Gentra Puregene Blood Kit (Qiagen, Hilden, Germany). DNA purity and concentration were confirmed using a NanoDrop ND-1000 spectrophotometer.

We selected the SNPs previously associated with connective tissue diseases. We chose genomic regions based on a review of the literature and used the most significant reported SNPs which had been analyzed in relatively large groups of cases. All polymorphisms selected for this study had minor allele frequencies > 0.4 to achieve enough statistical power. Altogether, two SNPs in *PPAR gamma-2* (rs1801282, rs3856806) and one in *β3-AR* gene (rs4994) were analyzed.

The SNPs were genotyped using predesigned TaqMan® SNP genotyping assays (Life Technologies, Carlsbad, California; assay IDs: *PPAR gamma-2* (rs1801282: C_1129864_10) and *β3-AR* gene (rs4994: C_2215549_20)). The polymerase chain reaction-restriction fragment length polymorphism (PCR-RFLP analysis) was performed with HOT FIREPol Probe qPCR Mix Plus (no ROX) according to the manufacturer's instructions provided by Solis BioDyne (Tartu, Estonia). The PCR thermal cycling was as follows: initial denaturation at 95°C for 15 min.; 40 cycles of 95°C for 15 sec and 60°C for 60 sec. Thermal cycling was performed using a CFX96 Touch™ Real-Time PCR Detection System (Bio-Rad, Hercules, California, U.S.). As a quality control measure, negative controls and approximately 5% of samples were genotyped in duplicate to check genotyping accuracy. The genotypes of selected samples were confirmed by direct sequencing (OLIGO, IBB, Warsaw, Poland).

In case of C1431T (rs3856806) in *PPAR gamma-2* genes, the PCR-restriction fragment length polymorphism (PCR-RFLP) method was applied. The 170 bp PCR product of exon 6 was digested with the Eco72I enzyme (according to the manufacturer's instructions: Fermentas, Vilnius, Lithuania). Digestion products were separated by 2.5% agarose gel electrophoresis. In the case of wild-type DNA, two bands of 127 bp and 43 bp were present. The wild-type form was not digested by this endonuclease. The genotypes of selected samples were confirmed by direct sequencing (OLIGO, IBB, Warsaw, Poland).

### 2.5. Statistical Analysis

GraphPad PRISM 5 Software (GraphPad, San Diego, CA) was used for statistical calculations. Genotype data were tested for deviations from the Hardy-Weinberg equilibrium. The chi-squared test was used to analyze the differences in genotype/allele frequencies between connective tissue disease (CTD) patients and the controls, as well as between normo- and hypertensive patients. The strength of associations between the *PPAR gamma-2* genotypes (rs1801282 and rs3856806) among studied groups and *ADRB3* gene (Trp64Arg) was calculated using logistic regression and expressed as an odds ratio (95% CI), and the differences were considered significant if the value of probability (*P*) was less than 0.05. Module contingency tables were used in these calculations. For polymorphisms, the wild-type or ancestral genotype/allele served as a reference category.

The distributions of the anthropometrical and biochemical data were tested with the Shapiro-Wilk normality test. If analyzed data were not normally distributed, nonparametric tests were used. Since the number of Ala12Ala homozygotes was small (in both CTD patients and the control group) compared to Pro12Pro homozygotes, they were calculated together with Pro12Ala heterozygotes for all the analyses and are presented as Ala12/X in Table 1. Similarly, patients with C1431T+T14131T polymorphisms were collapsed together and are presented as T1431/X, and in the same way, Trp64Arg+Arg64Arg polymorphisms were analyzed as Arg64/X.

Student's *t*-test was used to compare continuous variables between two groups if the data distribution was concordant with the normal distribution (Shapiro-Wilk test). If the data did not meet the criteria mentioned above, the nonparametric Mann-Whitney *U-*test was used. For normally distributed data, a multifactor ANOVA analysis was performed to determine whether the dependent variables were significantly different between study and control groups in relation to polymorphism and statin intake. Otherwise, the nonparametric Kruskal-Wallis test was used. A *P* value less than 0.05 was regarded as statistically significant. Statistical analyses were performed with STATISTICA 12 (including STATISTICA Medical Package 2.0; StatSoft, Inc. 2014 software) and SPSS 22 (IBM, Inc., Chicago, IL, USA).

## 3. Results

The analysis of allele and genotype frequencies showed no differences between CTD and control groups (Table 2).

Four different comparisons of anthropometric and blood pressure parameters in CTD vs. control group and hypertensive vs. normotensive subjects are shown in Table 3. The first comparison of patients with CTD and control group (Table 3 (I)) revealed that patients with CTD have a lower hip circumference, body fat and water, SBP, DBP, and MAP and a higher body water amount, serum glucose, and triglyceride level. The second comparison of hypertensive (*n* = 89) to normotensive (*n* = 118) patients (Table 3 (II)) demonstrated that hypertensive patients are older and have higher body mass, WC, LBM, body water content, BMI, glucose, and TG level.

The third analysis including only patients with CTD diseases (Table 3 (III)) indicated that hypertensive CTD patients (*n* = 56) are older and have a higher body mass, WC, body fat amount, LBM, body water content, BMI, and serum glucose level when compared to normotensive subjects (*n* = 48). The fourth comparison of hypertensive subjects to normotensive ones in the control group (Table 3 (IV)) showed that the groups differed in age and glucose level.

The multivariate analysis of all subjects in this study (Table 1 (I)) showed that hypertensive patients with Ala12/X or Trp64Trp genotypes have a higher body mass and waist circumference when compared to normotensive subjects. The levels of TG were higher in patients with Trp64Arg genotype, while subjects with T1431/X have higher WC. Similar relationships were observed in hypertensive CTD when compared to normotensive subjects with CTD (Table 1 (II)).

For results presented in Tables 1 and 3, adjustment for the Family-Wise Error Rate (FWER) in multiple comparisons was not calculated, because these corrections were not included in the primary hypothesis of the current study. The *P* values uncorrected for use of multiple comparisons were presented for illustrative purposes, without making a categorical assertion.

Gene-to-gene interaction in the context of WC and BMI in patients with hypertension and normal blood pressure was analyzed in the control and CTD groups using two-way ANOVA (Table 4).

## 4. Discussion

This study reveals associations between the analyzed polymorphisms and metabolic parameters and blood pressure characteristics in CTD. We have shown that hypertensive patients with the Ala12/X or Trp64Trp genotypes have an increased risk of visceral obesity development. A tendency to visceral fat distribution was also observed in hypertensive patients with CTD.

The data presented in Table 2 showed no differences between CTD and control groups. Since ethnic and environmental variations for the analyzed alleles have been reported, we compared the data to other Caucasian populations. The frequencies of all analyzed alleles were comparable in both groups (CTD and control groups as well as hypertensive and normotensive subjects). The analyzed frequency of the Ala12 allele carrier was similar to the allele frequencies in other Caucasian populations (0.11–0.13), including those of Polish ethnicity [2, 4, 25]. The frequencies of the T1431 and Arg64 allele carriers were also comparable to frequencies observed in Polish subjects (T1431 (frequency 0.148) and Arg64 (frequency 0.101)) [24].

The data in Table 3 showed different comparisons in the four groups of analyzed patients. In this study, we analyzed not only SBP and DBP but also parameters of hemodynamic characteristics such as MAP (which refers to the steady pressure and vascular resistance of small arteries) and PP (which is determined by stroke volume, arterial stiffness, and wave reflections) [20, 22]. The first comparison between CTD and the control group (Table 3 (I)) indicated that patients with CTD have proper blood pressure (SPB < 140 mmHg and DBP < 90 mmHg) and were characterized by lower MAP when compared to the control group. This could have been caused by hypotensive treatment modified at every admission to the hospital. Similar associations were observed when comparing hypertensive vs. normotensive patients with CTD (*P* = 0.0499) (Table 3 (III)). Unfortunately, low MAP is associated with a poorer prognosis and an 11% increased mortality in patients with cardiovascular diseases [26].

To compare body components between the analyzed groups, we used the bioimpedance method, which allows an estimation of lean body mass (free fat mass) and body fat content [27–29]. In our study, body mass, LBM, and BMI values were comparable between CTD and control groups. Similarly, other studies have not shown differences in fat-free mass (LBM) and fat mass between patients with rheumatoid arthritis and control groups if the groups have comparable BMI within the recommended range (<25 kg/m^2^) [30, 31]. But in our study, patients with CTD were characterized by higher water content, serum glucose, and triglyceride level when compared to healthy subjects. Changes in body compartments and metabolic parameters can be associated with an inflammatory state present in the course of autoimmune disorders as well as being a side effect of glucocorticosteroid use [32, 33].

The second comparison showed that hypertensive patients were older when comparing normotensive subjects independent of the analyzed groups (whole group (Table 3 (II)), CTD patients (Table 3 (III)), or control group (Table 3 (IV))). This fact reflects the general tendency of increasing blood pressure with age [34]. Moreover, both aging and hypertension have a critical role in cardiovascular and cerebrovascular complications [35]. In this study, hypertensive subjects had a higher body mass and WC (Tables 3 (II) and 3 (III)). These data are in accordance with the fact that the prevalence of hypertension increases with weight gain and the visceral distribution of body fat [36]. Moreover, hypertensive subjects have higher body water content, which could be associated with the tendency of water gathering in the hypertensive state [37]. The analysis (Table 3 (II)) of all hypertensive (*n* = 89) and all normotensive subjects (*n* = 118) did not reveal differences between SBP, DBP, PP, and MAP, because hypertensive subjects used medications to lower blood pressure; however, the analysis of blood pressure suggested the intensification of hypotensive therapy to achieve therapeutic goals mainly in the control group, because patients with CTD had proper blood pressure (Table 3 (I)).

The third comparison (Table 3 (III)) including only patients with CTD shows that hypertensive patients were older and had a higher body mass, BMI, WC, and body fat content; however, the value of PP was comparable in all the presented analyses. PP is considered a predictor of cardiovascular disorders in the general population [38] and hypertensive state [39]. The calculation of hemodynamic parameters, if the normal blood pressure of 120/80 mmHg is present, gives PP = 40 mmHg. Unfortunately, in this study, the value of PP was high and exceeded 54 mmHg in all analyzed subjects. Thus, elevated PP in patients with CTD increases the risk of cardiovascular disorders [38]. This fact can be explained by a reduction in cardiac output, which neurohumorally activates a compensatory mechanism and a systemic vascular resistance. In consequence, the arterial stiffness increases [38, 39]. In Table 3, chi-squared analysis for the number of patients with hypertension (*n* = 56, *n* = 33) and normal blood pressure (*n* = 48, *n* = 70) in CTD and control groups showed that the hypertensive state was significantly related to CTD (27.05%) while normotensive patients were predominantly present in the control group (33.82%; *P* = 0.0015).

Data in Table 1 show that hypertensive patients, carriers of Ala12 allele, have a higher body mass and WC, which reflects the tendency for the coexistence of this allele with increased blood pressure [9–11, 40, 41]. Moreover, the Ala12 allele is also associated with higher body mass and BMI value and a tendency to obesity not only in Caucasian subjects [25] but also in other populations [4, 5]. The Ala12 carrier is also related to increased body mass in women, and the additive effect of coexisting Ala12 and T1431 alleles is present [16]. In this study, patients with the T1431/X genotype were characterized by higher WC; however, we did not observe any additive effect of Ala12 or T1431 alleles (data not shown in tables). Interestingly, hypertensive homozygous subjects with the Trp64Trp genotype (both CTD and control groups) were characterized by a higher body mass, WC, and TG level when compared to normotensive subjects. In contrast to our study, Corella et al. reported that the Arg64 allele was associated with a higher BMI in a Mediterranean Spanish population [42]. We suspect that such differences are related to different ethnicities, which are related to different genetic and environmental factors and the presence of CTD.

Two-way ANOVA has been used to determined differences between values of WC and BMI in hypertensive and normotensive groups and analyzed genotypes in patients with CTD and control group (Table 4). This analysis showed that Ala12/X genotype determined the higher values of waist circumference in patients with hypertension and CTD (*P* = 0.0216).

## 5. Conclusion

We did not find differences between genotype/allele frequencies between the analyzed hypertensive patients with CTD diseases and the control group; however, we showed that the analyzed polymorphisms Pro12Ala, T14131/X, and Trp64Trp were associated with worse anthropometric parameters in hypertensive subjects. From the analyzed genetic variants, the Trp64Trp genotype shows the stronger relation with hypertension, because it is associated not only with a higher body mass and waist circumference but also with higher triglyceride levels and may predict the development of metabolic syndrome in the future. Moreover, the hypertensive state was related to higher age and tended to visceral fat distribution (higher body mass, BMI, and WC). Although the patients with CTD were characterized by proper values of SBP and DBP, the MAP was lower in this group. Hypertension was well treated in CTD patients, but the intensification of lowering blood pressure therapy is necessary in the control group. Our findings suggest complex genotype-environmental interactions with hypertensive risk, and further studies should show a more complex relationship between the analyzed polymorphisms and metabolic risk.

## Figures and Tables

**Table 1:** Genetic background of hypertension in analyzed groups.

Analyzed parameters	Group	Polymorphism	I. Both groups (CTD and control groups)			II. Patients with CTD		
			*N*	*X* ± SD	*P* value	*N*	*X* ± SD	*P* value
Body mass (kg)	Hypertensive	Ala12/X	28	78.17 ± 17.74	0.0061	15	81.17 ± 21.28	0.0169
	Normotensive		36	68.64 ± 11.42		15	67.91 ± 13.04	

WC (cm)	Hypertensive	Ala12/X	28	95.14 ± 19.11	0.0012	15	99.56 ± 23.31	0.0044
	Normotensive		36	83.77 ± 11.51		15	83.46 ± 11.05	

WC (cm)	Hypertensive	T1431/X	23	92.86 ± 21.24	0.0084	13	98.42 ± 24.75	0.0041
	Normotensive		37	83.05 ± 13.26		12	80.51 ± 12.07	

Body mass (kg)	Hypertensive	Trp64Trp	73	74.42 ± 12.05	0.0138	45	73.70 ± 12.27	0.0383
	Normotensive		105	69.22 ± 13.02		41	66.83 ± 13.95	

WC (cm)	Hypertensive	Trp64Trp	73	91.21 ± 14.28	0.0009	45	91.85 ± 15.75	0.0201
	Normotensive		105	84.12 ± 13.77		41	83.92 ± 15.60	

TG (mmol/L)	Hypertensive	Trp64Trp	73	1.82 ± 1.18	0.0030	45	2.10 ± 1.31	0.0224
	Normotensive		105	1.41 ± 0.78		41	1.55 ± 1.01	

WC: waist circumference; TG: triglycerides.

**Table 2:** Allele frequency distribution and logistic regression analysis (with odds ratio (OR) and 95% confidence interval (CI)) for the associations of the studied *PPAR gamma-2* and *ADRB3* polymorphisms with CTD vs. control group and hypertension (HA) vs. normal blood pressure.

*PPARγ* polymorphisms				
Alleles	*N* (frequency)		Logistic regression analysis	
rs1801282	CTD (*N* = 104)	Control group (*N* = 103)	OR (95% CI)	*P*
Pro	174 (0.84)	169 (0.82)	1^1^	
Ala	34 (0.16)	37 (0.18)	0.8925 (0.5351-1.489)	0.6629

	Hypertensive (*N* = 89)	Normotensive (*N* = 118)	OR (95% CI)	*P*
Pro	146 (0.82)	196 (0.83)	1^1^	
Ala	32 (0.18)	40 (0.17)	1.074 (0.6436-1.792)	0.7846

rs3856806	CTD	Controls	OR (95% CI)	*P*
C1431	181 (0.87)	168 (0.82)	1^1^	
T1431	27 (0.13)	38 (0.18)	0.6595 (0.3858-1.127)	0.1264

	Hypertensive	Normotensive	OR (95% CI)	*P*
C1431	153 (0.86)	196 (0.83)	1^1^	
T1431	25 (0.14)	40 (0.17)	0.8007 (0.4653-1.378)	0.4213

*ADRB3* polymorphism				
Alleles	*N* (frequency)		Logistic regression analysis	

rs4994	CTD	Controls	OR (95% CI)	*P*
Trp64	190 (0.91)	195 (0.95)	1^1^	
Arg64	18 (0.09)	11 (0.05)	1.679 (0.7726-3.651)	0.1865

	Hypertensive	Normotensive	OR (95% CI)	*P*
Trp64	162 (0.91)	223 (0.94)	1^1^	
Arg64	16 (0.09)	13 (0.06)	1.694 (0.7927-3.621)	0.1696

^1^Reference category; OR (95% CI): odds ratio (95% confidence interval).

**Table 3:** Comparison of anthropometric and blood pressure parameters in CTD vs. control group and hypertensive vs. normotensive subjects.

Analyzed parameters	I. CTD *n* = 104	Control *n* = 103		II. Hypertensive *n* = 89	Normotensive *n* = 118	
	*X* ± SD	*X* ± SD	*P*	*X* ± SD	*X* ± SD	*P*
Age (years)	53.76 ± 15.80	56.00 ± 3.91	0.1639	57.73 ± 12.50	52.72 ± 10.34	**0.0019**
Height (cm)	165.92 ± 9.67	164.40 ± 5.26	0.1611	165.74 ± 8.67	164.74 ± 7.10	0.3624
Body mass (kg)	70.45 ± 15.66	71.96 ± 12.02	0.4367	74.40 ± 14.95	68.79 ± 12.69	**0.0039**
WC (cm)	88.02 ± 15.81	86.14 ± 12.42	0.3420	90.82 ± 14.63	84.27 ± 13.29	**0.0009**
Hip circumference (cm)	98.31 ± 8.82	103.50 ± 8.82	**0.00001**	101.96 ± 9.43	100.09 ± 8.93	0.1464
Body fat (kg)	24.27 ± 11.20	28.38 ± 8.54	**0.0034**	27.40 ± 12.06	25.50 ± 8.41	0.1838
LBM (kg)	47.52 ± 13.53	47.08 ± 36.33	0.9076	51.97 ± 39.85	43.78 ± 9.42	**0.0323**
Body water (L)	36.06 ± 10.17	33.32 ± 4.75	**0.0138**	36.95 ± 10.54	32.99 ± 4.87	**0.0004**
BMI (kg/m^2^)	25.50 ± 4.69	26.59 ± 4.13	0.0763	26.97 ± 4.37	25.34 ± 4.39	**0.0085**
SBP (mmHg)	135.05 ± 16.77	142.26 ± 22.82	**0.0102**	140.97 ± 18.27	136.88 ± 21.59	0.1519
DBP (mmHg)	78.22 ± 11.38	87.80 ± 13.78	**0.00001**	84.32 ± 14.01	81.98 ± 13.04	0.2189
MAP (mmHg)	97.16 ± 11.74	105.95 ± 16.20	**0.00001**	103.20 ± 14.26	100.28 ± 15.08	0.1601
PP (mmHg)	56.83 ± 13.80	54.47 ± 13.06	0.2078	56.65 ± 13.21	54.90 ± 13.66	0.3547
Glucose (mmol/L)	5.40 ± 0.98	5.30 ± 0.67	**0.0001**	5.43 ± 1.06	5.29 ± 0.63	**0.00001**
TG (mmol/L)	1.74 ± 1.12	1.33 ± 0.63	**0.00001**	1.75 ± 1.10	1.38 ± 0.75	**0.0001**
Steroids (mg/day)	38.47 ± 41.44	—	—	42.21 ± 41.40	34.10 ± 41.50	0.3221
WHR	0.89 ± 0.15	0.83 ± 0.07	0.00001	0.89 ± 0.15	0.84 ± 0.09	**0.00001**

Analyzed parameters	III. CTD			IV. Control groups		
	Hypertensive *N* = 56	Normotensive *N* = 48		Hypertensive *N* = 33	Normotensive *N* = 70	
	*X* ± SD	*X* ± SD	*P*	*X* ± SD	*X* ± SD	*P*
Age (years)	57.93 ± 15.53	48.90 ± 14.84	**0.0032**	57.39 ± 3.90	55.34 ± 3.77	**0.0123**
Height (cm)	165.71 ± 10.09	166.18 ± 9.24	0.8055	165.79 ± 5.64	163.75 ± 4.97	0.0654
Body mass (kg)	74.39 ± 16.50	65.85 ± 13.38	**0.0050**	74.43 ± 12.12	70.80 ± 1.88	0.1533
WC (cm)	91.56 ± 15.96	83.90 ± 14.74	**0.0130**	89.56 ± 12.17	84.53 ± 12.29	0.0546
Hip (cm)	99.76 ± 8.83	96.63 ± 8.60	0.0705	105.70 ± 9.38	102.46 ± 8.42	0.0827
Body fat (kg)	26.31 ± 13.25	21.89 ± 7.68	**0.0444**	29.25 ± 9.63	27.98 ± 8.02	0.4841
LBM (kg)	50.28 ± 15.28	44.31 ± 10.40	**0.0241**	54.84 ± 62.86	43.42 ± 8.75	0.1376
Body water (L)	38.39 ± 12.26	33.35 ± 6.07	**0.0110**	34.52 ± 6.13	32.75 ± 3.86	0.0776
BMI (kg/m^2^)	26.95 ± 4.69	23.81 ± 4.13	0.0004	27.01 ± 3.83	26.39 ± 4.27	0.4827
SBP (mmHg)	137.66 ± 16.75	132.00 ± 16.44	0.0861	146.58 ± 19.62	140.23 ± 24.05	0.1891
DBP (mmHg)	80.04 ± 12.75	76.10 ± 9.23	0.0790	91.58 ± 13.19	86.01 ± 13.78	0.0556
MAP (mmHg)	99.24 ± 12.56	94.74 ± 10.29	**0.0499**	109.91 ± 14.64	104.09 ± 16.65	0.0887
PP (mmHg)	57.63 ± 14.08	55.90 ± 13.56	0.5269	55.00 ± 11.58	54.21 ± 13.78	0.7773
Glucose (mmol/L)	5.47 ± 1.18	5.34 ± 0.69	**0.0002**	5.37 ± 0.82	5.26 ± 0.59	**0.0229**
TG (mmol/L)	1.96 ± 1.22	1.49 ± 0.95	0.0791	1.38 ± 0.73	1.30 ± 0.57	0.0921
Steroids (mg/day)	42.21 ± 41.40	34.10 ± 41.50	0.3221	—	—	—
WHR	0.92 ± 0.17	0.86 ± 0.11	0.0734	0.84 ± 0.08	0.82 ± 0.07	0.1582

WC: waist circumference; LBM: lean body mass; BMI: body mass index; SBP: systolic blood pressure; DBP: diastolic blood pressure; MAP: mean arterial pressure; PP: pulse pressure; TG: triglycerides; WHR: waist to hip ratio.

**Table 4:** Gene-to-gene interaction in hypertensive and normotensive groups with CTD and control groups.

Group	Genotype	CTD			Control		
		*X* ± SD	*n*	*P*	*X* ± SD	*n*	*P*
WC							
Normotensive	Pro12Pro	84.09 ± 16.30	33	ns	84.76 ± 12.49	49	ns
	Ala12/X	83.47 ± 11.05	15		84.00 ± 12.10	21	
Hypertensive	Pro12Pro	88.63 ± 11.27	41	0.0216	89.25 ± 12.80	20	ns
	Ala12/X	99.57 ± 23.31	15		90.04 ± 11.63	13	
Normotensive	C1431C	85.03 ± 15.51	36	ns	84.67 ± 11.49	45	ns
	T1431/X	80.50 ± 12.08	12		84.28 ± 13.87	25	
Hypertensive	C1431C	89.49 ± 11.81	43	ns	91.26 ± 11.41	23	ns
	T1431/X	98.42 ± 24.75	13		85.65 ± 13.56	10	
Normotensive	Trp64Trp	83.93 ± 15.60	41	ns	84.25 ± 12.60	64	ns
	Arg64/X	83.71 ± 8.94	7		87.50 ± 8.50	6	
Hypertensive	Trp64Trp	91.86 ± 15.76	45	ns	90.20 ± 11.73	28	ns
	Arg64/X	90.36 ± 17.49	11		86.00 ± 15.39	5	

BMI (kg/m^2^)							
Normotensive	Pro12Pro	23.76 ± 4.62	33	ns	26.64 ± 4.64	49	ns
	Ala12/X	23.90 ± 2.94	15		25.84 ± 3.30	21	
Hypertensive	Pro12Pro	25.37 ± 4.78	41	ns	26.87 ± 4.18	20	ns
	Ala12/X	28.76 ± 5.89	15		27.24 ± 3.84	13	
Normotensive	C1431C	23.98 ± 4.49	36	ns	26.49 ± 3.88	45	ns
	T1431/X	23.29 ± 2.96	12		26.23 ± 4.99	25	
Hypertensive	C1431C	25.02 ± 4.49	43	ns	27.28 ± 3.99	23	ns
	T1431/X	27.64 ± 4.71	13		26.40 ± 3.57	10	
Normotensive	Trp64Trp	24.13 ± 4.30	41	ns	26.49 ± 4.40	64	ns
	Arg64/X	21.88 ± 2.42	7		25.41 ± 2.67	6	
Hypertensive	Trp64Trp	26.96 ± 3.84	45	ns	27.32 ± 3.73	28	ns
	Arg64/X	27.08 ± 7.38	11		25.28 ± 4.42	5	

WC: waist circumference; BMI: body mass index; CTD: connective tissue diseases.

## Data Availability

The association study data used to support the findings of this study are included within the article.

## References

[1] Sharma A. M., Staels B. (2007). Peroxisome proliferator-activated receptor *γ* and adipose Tissue—Understanding obesity-related changes in regulation of lipid and glucose metabolism. *The Journal of Clinical Endocrinology and Metabolism*.

[2] Grygiel-Gorniak B., Mosor M., Marcinkowska J., Przyslawski J., Nowak J. (2016). Impact of the PPAR gamma-2 gene polymorphisms on the metabolic state of postmenopausal women. *Journal of Biosciences*.

[3] Grygiel-Górniak B. (2014). Peroxisome proliferator-activated receptors and their ligands: nutritional and clinical implications-a review. *Nutrition Journal*.

[4] Ghoussaini M., Meyre D., Lobbens S. (2005). Implication of the Pro12Ala polymorphism of the *PPAR-gamma* 2gene in type 2 diabetes and obesity in the French population. *BMC Medical Genetics*.

[5] Passaro A., Dalla Nora E., Marcello C. (2011). PPAR*γ* Pro12Ala and ACE ID polymorphisms are associated with BMI and fat distribution, but not metabolic syndrome. *Cardiovascular Diabetology*.

[6] Doney A. S. F., Fischer B., Cecil J. E. (2004). Association of the Pro12Ala and C1431T variants of *PPARG* and their haplotypes with susceptibility to type 2 diabetes. *Diabetologia*.

[7] Dong C. P., He L., Li J. N., Ye F., He M., Wang Y. (2008). Association of Prol2Ala and C1431T polymorphism of the PPAR-*γ*2 gene and their haplotypes with obesity and type 2 diabetes. *Chinese Journal of Medical Genetics*.

[8] Jaziri R., Lobbens S., Aubert R. (2006). The *PPARG* Pro12Ala polymorphism is associated with a decreased risk of developing hyperglycemia over 6 years and combines with the effect of the *APM1* G-11391A single nucleotide polymorphism: the Data From an Epidemiological Study on the Insulin Resistance Syndrome (DESIR) study. *Diabetes*.

[9] Östgren C., Lindblad U., Melander O., Melander A., Groop L., Råstam L. (2003). Peroxisome proliferator-activated receptor-*γPro12Ala* polymorphism and the association with blood pressure in type 2 diabetes: skaraborg hypertension and diabetes project. *Journal of Hypertension*.

[10] Kim K., Lee S., Valentine R. J. (2007). Association of pro12Ala polymorphism in the peroxisome proliferative-activated receptor gamma2 gene with obesity and hypertension in Korean women. *Journal of Nutritional Science and Vitaminology*.

[11] Lu Z., Dong B., Mo X. (2008). Pro12Ala polymorphism in PPAR gamma 2 associated with essential hypertension in Chinese nonagenarians/centenarians. *Experimental Gerontology*.

[12] Evans D., de Heer J., Hagemann C. (2001). Association between the P12A and c1431t polymorphisms in the peroxisome proliferator activated receptor *γ* (PPAR*γ*) gene and type 2 diabetes. *Experimental and Clinical Endocrinology & Diabetes*.

[13] Leibovitz E., Schiffrin E. L. (2005). The many faces of PPARgamma. *Cell*.

[14] Leibovitz E., Schiffrin E. L. (2007). PPAR activation: a new target for the treatment of hypertension. *Journal of Cardiovascular Pharmacology*.

[15] Zhou X., Chen J., Xu W. (2012). Association between C1431T polymorphism in peroxisome proliferator-activated receptor-*γ* gene and coronary artery disease in Chinese Han population. *Molecular Biology Reports*.

[16] Valve R., Sivenius K., Miettinen R. (1999). Two polymorphisms in the peroxisome proliferator-activated receptor-gamma gene are associated with severe overweight among obese women. *The Journal of Clinical Endocrinology and Metabolism*.

[17] Knoblauch H., Busjahn A., Muller-Myhsok B. (1999). Peroxisome proliferator-activated receptor *γ* gene locus is related to body mass index and lipid values in healthy nonobese subjects. *Arteriosclerosis, Thrombosis, and Vascular Biology*.

[18] Grygiel-Górniak B., Ziółkowska-Suchanek I., Kaczmarek E., Mosor M., Nowak J., Puszczewicz M. (2018). PPARgamma-2 and ADRB3 polymorphisms in connective tissue diseases and lipid disorders. *Clinical Interventions in Aging*.

[19] WHO, World Health Organization (2003). Diet, nutrition and the prevention of chronic diseases. *WHO Technical Report Series 916 chapter 5*.

[20] O'Brien E., Asmar R., Beilin L. (2003). European Society of Hypertension recommendations for conventional, ambulatory and home blood pressure measurement. *Journal of Hypertension*.

[21] Nichols W. W., O’Rourke M. F., Arnold E. (2006). *Theoretical, Experimental and Clinical Principles*.

[22] Avolio A. P., van Bortel L. M., Boutouyrie P. (2009). Role of pulse pressure amplification in arterial hypertension: experts’ opinion and review of the data. *Hypertension*.

[23] Friedewald W. T., Levy R. I., Fredrickson D. S. (1972). Estimation of the concentration of low-density lipoprotein cholesterol in plasma, without use of the preparative ultracentrifuge. *Clinical Chemistry*.

[24] Grygiel-Górniak B., Kaczmarek E., Mosor M., Przysławski J., Nowak J. (2015). Association of PPAR-*γ*2 and *β*3-AR polymorphisms with postmenopausal hypertension. *Journal of Clinical Hypertension*.

[25] Beamer B. A., Yen C. J., Andersen R. E. (1998). Association of the Pro12Ala variant in the peroxisome proliferator-activated receptor-gamma2 gene with obesity in two Caucasian populations. *Diabetes*.

[26] Domanski M. J., Mitchell G. F., Norman J. E., Exner D. V., Pitt B., Pfeffer M. A. (1999). Independent prognostic information provided by sphygmomanometrically determined pulse pressure and mean arterial pressure in patients with left ventricular dysfunction. *Journal of the American College of Cardiology*.

[27] Johnson P. R., Greenwood M. R. C., Weiss L. (1988). The adipose tissue. *Cell and Tissue Biology: A Textbook of Histology*.

[28] Kyle U. G., Piccoli A., Pichard C. (2003). Body composition measurements: interpretation finally made easy for clinical use. *Current Opinion in Clinical Nutrition and Metabolic Care*.

[29] Jaffrin M. Y. (2009). Body composition determination by bioimpedance: an update. *Current Opinion in Clinical Nutrition and Metabolic Care*.

[30] Oranskiy S. P., Yeliseyeva L. N., Tsanaeva A. V., Zaytseva N. V. (2012). Body composition and serum levels of adiponectin, vascular endothelial growth factor, and interleukin-6 in patients with rheumatoid arthritis. *Croatian Medical Journal*.

[31] Akar S., Sarı İ., Çömlekci A. (2014). Body composition in patients with rheumatoid arthritis is not different than healthy subjects. *European Journal of Rheumatology*.

[32] Aronow W. S. (2017). Drug-induced causes of secondary hypertension. *Annals of Translational Medicine*.

[33] Yelnik C. M., Richey M., Haiduc V., Everett S., Zhang M., Erkan D. (2017). Cardiovascular disease prevention counseling program for systemic lupus erythematosus patients. *Arthritis Care & Research*.

[34] Virdis A., Bruno R. M., Fritsch Neves M., Bernini G., Taddei S., Ghiadoni L. (2011). Hypertension in the elderly: an evidence-based review. *Current Pharmaceutical Design*.

[35] Higashi Y., Kihara Y., Noma K. (2012). Endothelial dysfunction and hypertension in aging. *Hypertension Research*.

[36] Sironi A. M., Gastaldelli A., Mari A. (2004). Visceral fat in Hypertension: influence on insulin resistance and *β*-cell function. *Hypertension*.

[37] Coffman T. M. (2011). Under pressure: the search for the essential mechanisms of hypertension. *Nature Medicine*.

[38] Franklin S. S., Khan S. A., Wong N. D., Larson M. G., Levy D. (1999). Is pulse pressure useful in predicting risk for coronary heart disease: the Framingham Heart Study. *Circulation*.

[39] Fang J., Madhavan S., Cohen H., Alderman M. H. (1995). Measures of blood pressure and myocardial infarction in treated hypertensive patients. *Journal of Hypertension*.

[40] Douglas J. A., Erdos M. R., Watanabe R. M. (2001). The peroxisome proliferator-activated receptor-gamma2 Pro12A1a variant: association with type 2 diabetes and trait differences. *Diabetes*.

[41] Stefański A., Majkowska L., Ciechanowicz A. (2006). Association between the Pro12Ala variant of the peroxisome proliferator- activated receptor-gamma2 gene and increased 24-h diastolic blood pressure in obese patients with type II diabetes. *Journal of Human Hypertension*.

[42] Corella D., Guillén M., Portolés O. (2001). Gender specific associations of the Trp64Arg mutation in the *β*_3_-adrenergic receptor gene with obesity-related phenotypes in a Mediterranean population: interaction with a common lipoprotein lipase gene variation. *Journal of Internal Medicine*.

